# Bronchoscope: an important and easily overlooked vector of clinical infection with *Klebsiella pneumoniae*

**DOI:** 10.3389/fcimb.2026.1850121

**Published:** 2026-06-05

**Authors:** Wei Wang, Lijuan Qi, Xinying Wang, Tingchao Wu, Ren Ren, Shenyun Cao

**Affiliations:** 1 Intensive Care Department, The Affiliated Taian City Central Hospital of Qingdao University, Taian, China; 2 Department of Hematology, The Affiliated Taian City Central Hospital of Qingdao University, Taian, China; 3 Department of Laboratory Medicine, The Affiliated Taian City Central Hospital of Qingdao University, Taian, China

**Keywords:** biofilm, bronchoscope, carbapenem resistance, Klebsiella pneumoniae, transmission route

## Abstract

**Objective:**

To investigate the role of bronchoscopes in a Pediatric Intensive Care Unit (PICU) carbapenem-resistant *Klebsiella pneumoniae* (CRKP) outbreak and analyze the biological characteristics of the isolated strains, providing evidence for nosocomial infection prevention.

**Methods:**

A CRKP infection outbreak in a PICU was investigated. Four CRKP strains were isolated from the bronchoalveolar lavage fluid of infected patients, and one CRKP strain was isolated from the shared bronchoscope used for these patients. Antimicrobial susceptibility testing was performed to determine the drug resistance profile of the strains. Genotypic analysis was conducted to detect the presence of antimicrobial resistance genes and virulence-associated genes. Average nucleotide identity (ANI) and sequence typing (ST) were used to assess the homology of the five isolated CRKP strains. Silver staining was adopted to compare the biofilm formation capabilities of the bronchoscope-isolated strain, patient-isolated strains and the standard control strain.

**Results:**

All 5 CRKP isolates were fully resistant to cephalosporins, carbapenems and other agents, but susceptible to levofloxacin, trimethoprim/sulfamethoxazole and tigecycline, and all carried *bla*_NDM-1_, *mrkD* and other resistance/virulence genes. Homology analysis confirmed all strains belonged to the same clonal lineage (ST1697, ANI 99.97%–99.99%). The bronchoscope-isolated strain had the strongest biofilm-forming capacity, followed by patient isolates, all superior to the standard control strain.

**Conclusion:**

Contaminated bronchoscopes are the transmission vector of this monoclonal CRKP outbreak in PICU. The enhanced biofilm-forming ability of ST1697 CRKP facilitates its persistence and transmission, highlighting the necessity of strict adherence to bronchoscope disinfection and quality control to prevent hospital-acquired infections.

## 1 Introduction

*Klebsiella pneumoniae* (*K. pneumoniae*) is a significant opportunistic, gram-negative pathogen belonging to the *Enterobacteriaceae* family. This bacterium is a leading cause of both community-acquired and healthcare-associated infections, particularly in immunocompromised, elderly, or chronically ill individuals (Yang et al., 2022). *K. pneumoniae* is implicated in a spectrum of severe diseases, including pneumonia, bloodstream infections, urinary tract infections, and meningitis. The emergence and global spread of hypervirulent and multidrug-resistant strains, especially carbapenem-resistant *K. pneumoniae* (CRKP), have rendered some infections extremely difficult to treat, leading to high mortality and posing a critical public health challenge (Lei et al., 2024).

Environmental reservoirs play a crucial role in the spread of nosocomial infections caused by *K. pneumoniae*. A key factor enabling its survival in the environment is its capacity to form robust biofilms. Biofilms are structured communities of bacterial cells encased in a self-produced extracellular polymeric substance (EPS) (Centeleghe et al., 2023). This biofilm significantly enhances *K. pneumoniae*’s tolerance to desiccation, disinfectants, and antibiotics, compared to its planktonic counterparts. Current researches focus on elucidating the genetic regulators and EPS composition involved in biofilm formation. Understanding these mechanisms is vital for developing novel strategies (Arvand et al., 2026; Brunke et al., 2022). Many researches have demonstrated that *K. pneumoniae* can be also detected in hospital air and water systems, inanimate surfaces such as hospital bed rails, door handles, bedside tables, and shared medical equipment can become contaminated and serve as persistent sources of nosocomial outbreaks (Bereanu et al., 2024). Bronchoscope, as a common device for observing and sampling lung diseases, is widely utilized in hospitals. However, until now, the impact of bronchoscopy procedures on the transmission of *K. pneumoniae* may have been underestimated. The complex internal channels of reusable bronchoscopes may serve as potential reservoirs for *K. pneumoniae* biofilms, particularly in high-risk units such as the pediatric intensive care unit (PICU). The present study aimed to investigate a CRKP outbreak in a PICU, with a specific focus on evaluating the role of a shared bronchoscope as a potential transmission vector. We further sought to characterize the antimicrobial resistance profiles, clonal relatedness, and biofilm-forming capacities of the isolated strains to provide evidence-based recommendations for nosocomial infection prevention.

## 2 Materials and methods

### 2.1 Sample collection and the isolation of carbapenem-resistant *K. pneumoniae* strains

The sample collection took place in Taian City Central Hospital affiliated with Qingdao University. From October 20 to November 8, 2021, patients in the PICU ward of this hospital developed respiratory symptoms such as coughing and fever. We detected and isolated four strains of carbapenem-resistant *K. pneumoniae* (CRKP) from the bronchoalveolar lavage fluid of four patients. Importantly, all patients had used the same bronchoscope, one CRKP strain was isolated from the inner surface of the bronchoscope. The standard strain used was *K. pneumoniae* ATCC 700603.

### 2.2 The identification and antimicrobial susceptibility testing of *K. pneumoniae* strains

Bacterial identification was performed by WalkAway 96 PLUSNC50 combo panel (Beckman, United States) following the instructions of the manufacturer. Antimicrobial susceptibility testing was performed by both methods: the sensitivities of cefepime, cefoperazone/sulbactam, amoxicillin/clavulanic acid, ertapenem, ciprofloxacin, levofloxacin, cefazolin, cefotaxime were determined by the disk diffusion method, and the sensitivity of other antimicrobial agents was detected using the WalkAway 96 PLUS-NC50 combo panel. Antimicrobial susceptibility testing results were interpreted according to the FDA-identified interpretive criteria (U.S. Food and Drug Administration, 2023) criteria for tigecycline, and the CLSI M100-S35 (Clinical and Laboratory Standards Institute (CLSI), 2025) criteria for the remaining antibiotics.

### 2.3 Silver staining method

A single colony of *K. pneumoniae* was inoculated into LB liquid medium and cultured in an incubator at 37°C with shaking at 180 rpm overnight (approximately 16 h). A 2 cm × 2 cm × 1.5 mm silicone membrane was placed in each well of a 6-well plate. The overnight culture was adjusted to 0.5 McFarland standard and diluted 1:50 with LB liquid medium. Then, 2 mL of the diluted bacterial suspension was added and plate was incubated without shaking at 37°C for 48 h to allow biofilm formation on the silicone membrane surface. Then, bacteria adhering to the silicone membrane formed a biofilm. The silicone membranes were washed twice with PBS to remove planktonic bacteria, followed by fixation with pre-cooled 2.5% glutaraldehyde for 1 h. After two additional PBS washes, the membranes were treated with saturated calcium chloride solution for 15 min and washed again with PBS. Subsequently, the membranes were stained with 5% silver nitrate solution for 15 min, followed by treatment with 1% hydroquinone colloidal solution for 2 min and washing. Finally, the membranes were fixed with 5% sodium thiosulfate solution for 2 min, washed with PBS. After air-drying, the membranes were mounted on glass slides for observation and photography.The obtained images were analyzed using ImageJ software (version 1.54) to measure the stained area and calculate the percentage of biofilm coverage area relative to the total membrane surface area (Schneider et al., 2012). Three independent replicates were performed for each strain. Inter-group comparisons were conducted using one-way ANOVA followed by Tukey’s multiple comparisons test with GraphPad Prism 10 software. A P value < 0.05 was considered statistically significant.

### 2.4 The homology analysis of the *K. pneumoniae* strains

The isolated *K. pneumoniae* strains were sent for whole genome sequencing to the Biozeron biotechnology Co. Ltd (Shanghai, China) to identify clonal lineages. Isolates were sequenced on an Illumina MiSeq platform (Illumina, Inc., San Diego, USA). The raw paired-end reads were trimmed and quality controlled by Trimmomatic 0.36 (Bolger et al., 2014) with default parameters. Clean data obtained by the above quality control processes were subsequently used for *de novo* genome assembly using ABySS 2.2.0 (Simpson et al., 2009) with multiple-k-mer parameters. GapCloser was subsequently applied to fill the remaining local inner gaps and correct single base errors for the final assembly results (Xu et al., 2020). The resistance and virulence genes of strains were annotated in the Comprehensive Antibiotic Resistance Database (CARD) (https://card.mcmaster.ca/) and the Virulence Factors of Pathogenic Bacteria (VFDB) (http://www.mgc.ac.cn/VFs/links.htm), respectively. Genes with more than 95% identity were considered positive. Multilocus sequence types of *Klebsiella* spp. were identified in silico using MLST 2.0 provided by the Center for Genomic Epidemiology (https://pathogen.watch/). Average Nucleotide Identity (ANI) analysis was carried out using the JSpeciesWS server (https://jspecies.ribohost.com/jspeciesws) (Richter et al., 2016).

## 3 Results

### 3.1 The results of antimicrobial susceptibility testing, antibiotic resistance and virulence-associated genes

The antimicrobial susceptibility testing was conducted on five *K. pneumoniae* strains, the results were showed in **Table 1**, all strains exhibited 100% resistance to cephalosporins, cephamycins, aztreonam, carbapenems, and enzyme inhibitors. Resistance rates varied among aminoglycosides, 100% resistance to tobramycin, but 0 resistance to gentamicin and amikacin. Additionally, the isolates demonstrated good susceptibility to levofloxacin, trimethoprim/sulfamethoxazole, and tigecycline, with 0 resistance rates for all three agents. We further analyzed the antimicrobial resistance genes (ARGs) carried by the strains. The results showed that five *K. pneumoniae* strains all carried multiple drug resistance genes, including β-lactamase resistance genes (***bla*_NDM-1_**, ***bla****_VEB-3_*, and ***bla*_DHA-1_***)*, fluoroquinolone resistance genes (q*nrS1*, q*nrB4*, o*qxA*, o*qxB*) and aminoglycoside resistance genes(*aac(6’)-Ib-Hangzhou, catB8)*.(**Table 2**).

**Table 1:** The phenotype of drug sensitive test of *K. pneumonia* strains.

Strain	MIC (mg/L)														KB(mm)								
	CXM	FOX	CAZ	CRO	ATM	TZP	IPM	MEM	AMK	GEN	TOB	SXT	TGC	SAM	AMC	SCF	CTX	CZL	FEP	ETP	LVX	CIP	CZA
KP-2110299021	>16	>16	>16	>32	>16	>64	8	>8	≤4	≤2	>8	≤2/38	≤2	≥16/8	6	6	6	6	6	6	23	26	15
KP-2111019046	>16	>16	>16	>32	>16	>64	8	>8	≤4	≤2	>8	≤2/38	≤2	≥16/8	6	6	6	6	6	6	18	15	15
KP-2111039028	>16	>16	>16	>32	>16	>64	8	>8	≤4	≤2	>8	≤2/38	≤2	≥16/8	6	6	6	6	12	6	18	15	15
KP-2111039005	>16	>16	>16	>32	>16	>64	8	>8	≤4	≤2	>8	≤2/38	≤2	≥16/8	6	6	6	6	12	6	19	15	15
KP-21800014C	>16	>16	>16	>32	>16	>64	8	>8	≤4	≤2	>8	≤2/38	≤2	≥16/8	6	6	6	6	12	6	19	18	15

CXM, Cefuroxime; FOX, Cefoxitin; CAZ, Ceftazidime; CRO, Ceftriaxone; ATM, Aztreonam; TZP, Piperacillin/Tazobactam; IPM, Imipenem; MEM, Meropenem; AMK, Amikacin; GEN, Gentamicin; TOB, Tobramycin; SXT, Trimethoprim/Sulfamethoxazole; TGC, Tigecycline; SAM, Ampicillin/Sulbactam; AMC, Amoxicillin/Clavulanate; SCF, Cefoperazone/Sulbactam; CTX, Cefotaxime; CZL, Cefazolin; FEP, Cefepime; ETP, Ertapenem; LVX, Levofloxacin; CIP, Ciprofloxacin; CZA, Ceftazidime/Avibactam. Green-shaded: resistant phenotype. Light red-shade: susceptible. Yellow-shaded: intermediate.

**Table 2:** Antimicrobial resistance gene profiles of five *Klebsiella pneumoniae* isolates.

Strain	*Quinolone*				*Carbapenem and broad−spectrum β−lactam*				Aminoglycoside				Other	
	*oqxA*	*oqxB*	*qnrS1*	*qnrB4*	*bla*_VEB-3_	*bla*_NDM-1_	*bla*_DHA-1_	*bla*_OKP-B-10_	*aac(6’)-Ib-Hangzhou*	*catB8*	*aph(6)-Id*	*aph(3’’)-Ib*	*fosA*	*sul1*
KP-21800014C	+	+	+	+	+	+	+	+	+	+	+	+	+	+
KP-2110299021	+	+	+	+	+	+	+	–	+	+	–	–	+	+
KP-2111019046	+	+	+	+	+	+	+	+	+	+	+	+	+	+
KP-2111039005	+	+	+	+	+	+	+	–	+	+	–	–	+	+
KP-2111039028	+	+	+	+	+	+	+	–	+	+	–	–	+	+

+, presence of the gene; −, absence of the gene.

All five *Klebsiella pneumoniae* isolates exhibited identical virulence gene profiles. A variety of virulence genes associated with adhesion and fimbriae, iron acquisition, type VI secretion system, capsule synthesis, and transcriptional regulation were detected in all isolates. No differences in virulence gene carriage were observed among the five isolates. (**Table 3**).

**Table 3:** Virulence gene profiles of five *K. pneumoniae* isolates.

VF class	Virulence genes	All 5 isolates
Adhesion and Fimbriae	fimA, fimB, fimC, fimD, fimE, fimF, fimG, fimH, fimI, fimK, mrkA, mrkB, mrkC, mrkD, mrkF, mrkH, mrkI, mrkJ, yagZ/ecpA, yagY/ecpB, yagX/ecpC, yagW/ecpD, ykgK/ecpR	+
Iron Acquisition	entA, entB, entC, entD, entE, entF, fepA, fepB, fepC, fepD, fepG, fes, iroE, ybdA	+
Type VI Secretion System	tli1, sciN/tssJ, tssF, tssG, impA/tssA, vipA/tssB, vipB/tssC, vasE/tssK, dotU/tssL, hcp/tssD, clpV/tssH, vgrG/tssI	+
Capsule Synthesis	galF, cpsACP, wzi, ugd, manB, manC, gnd	+
Regulation	rcsA, rcsB	+

+, presence of the gen.e.

### 3.2 The homologous analysis of *K. pneumoniae* strains

Sequencing analysis of the five *K. pneumoniae* strains isolated in this study revealed that all were ST1697. Homology analysis showed ANI values ranging from 99.97% to 99.99% among the five strains, indicating they belonged to the same clonal lineage. This indicates that bronchoscopes serve as a significant transmission vector for *K. pneumoniae*, playing a crucial role in the hospital-acquired infections.

### 3.3 The detection of *K. pneumoniae* strain biofilm

**Figure 1:**
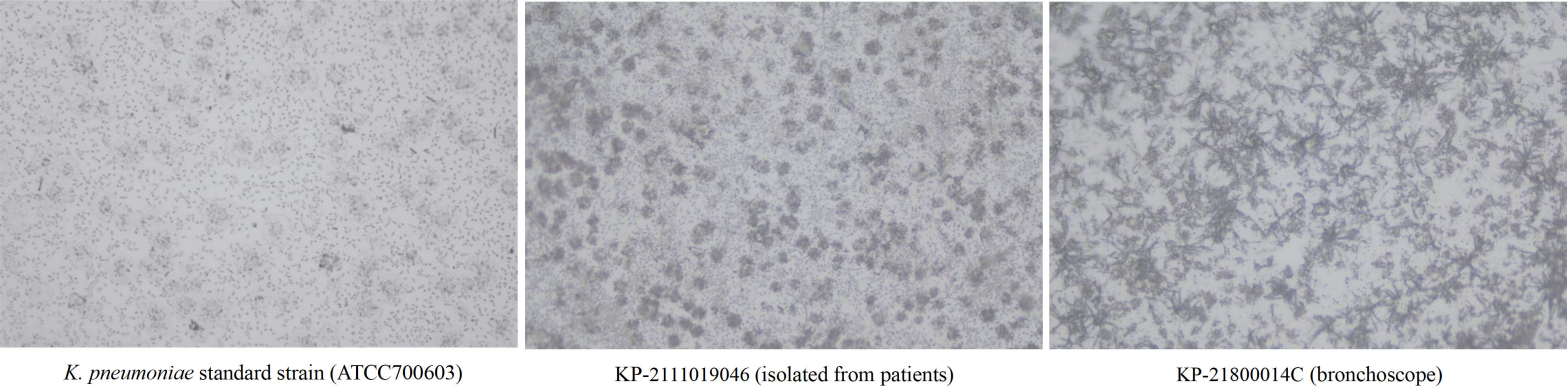
The sliver staining results of *K. pneumoniae* biofilms.

**Figure 2:**
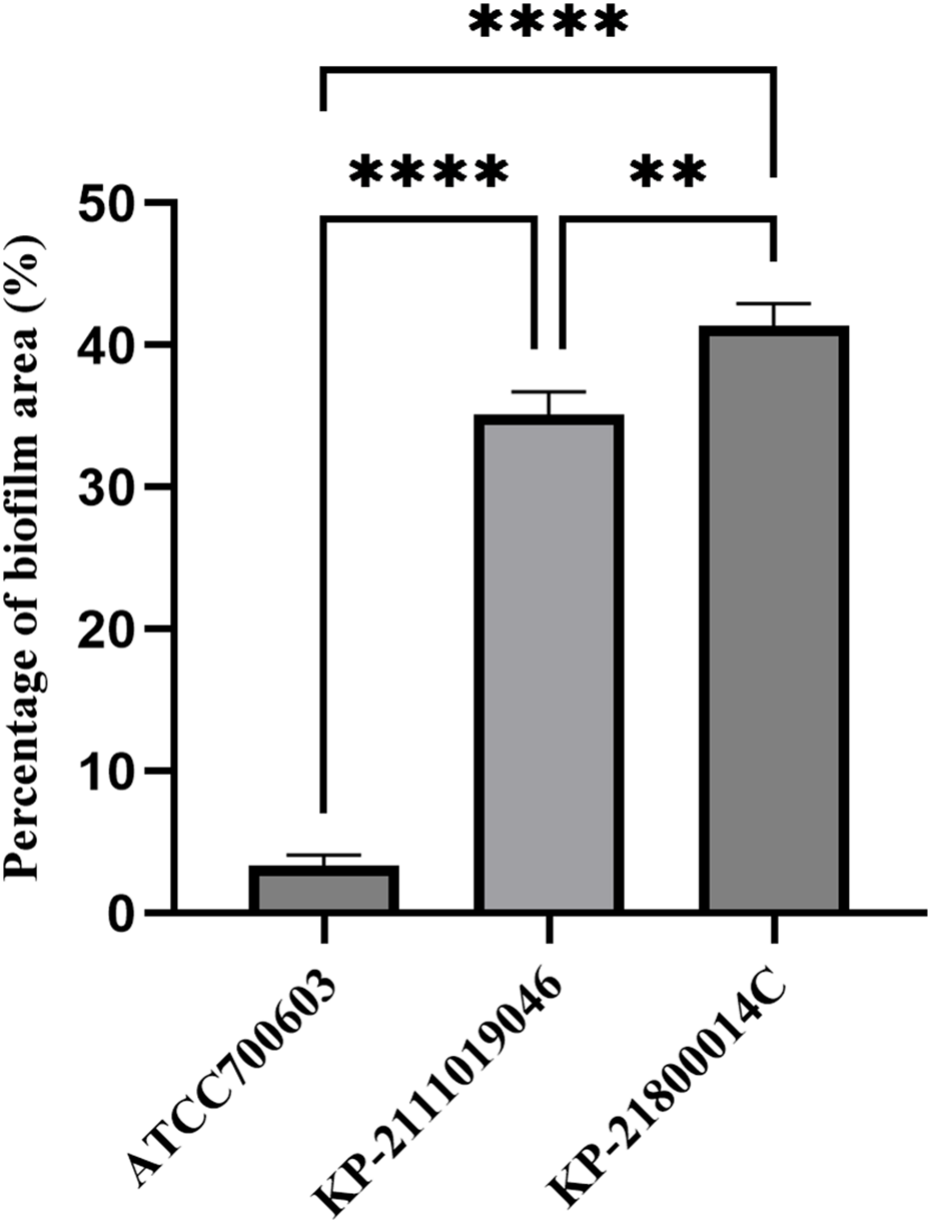
Quantitative analysis of biofilm formation capacity among *K. pneumoniae* strains The percentage of biofilm coverage area was measured by ImageJ software following silver staining. Data are presented as mean ± SD from three independent replicates. ****P < 0.0001, **P = 0.0035. ATCC 700603, standard control strain; KP-2111019046, patient-derived strain; KP-21800014C, bronchoscope-derived strain.

Silver staining was used to detect bacterial biofilms of CRKP strains from bronchoscopes and patients. Results showed that the CRKP biofilm from bronchoscopes exhibited distinct black, large-clump aggregates, while the biofilm from patients displayed distinct black, small-clump aggregates. In contrast, the standard strain showed scattered black dots or blank areas (**Figure 1**). Quantitative image analysis revealed that the percentage of biofilm coverage area was significantly higher in the bronchoscope-derived strain (41.3 ± 1.6%) compared to the patient-derived strain (35.1 ± 1.6%) and the standard strain (3.4 ± 0.7%). All pairwise comparisons showed statistically significant differences (bronchoscope-derived vs. patient-derived, P = 0.0035; bronchoscope-derived vs. standard, P < 0.0001; patient-derived vs. standard, P < 0.0001) (**Figure 2**).

## 4 Discussion

The five *K. pneumoniae* isolates in this study exhibited 100% resistance to carbapenem antibiotics, consistent with national surveillance trends-CHINET monitoring revealed that in 2025, resistance rates of *K. pneumoniae* to meropenem and imipenem in China had risen to 21.9% and 21.0%, respectively (China Antimicrobial Surveillance Network, 2025). Resistance gene analysis revealed that all isolates carried multiple drug resistance genes. The coexistence of *NDM-1* and *VEB-3* explains the high resistance of isolated strains to nearly all β-lactam antibiotics, including carbapenems (Naas et al., 2006; Nordmann et al., 2011). However, the strains in this study exhibited a unique inconsistent resistance pattern to aminoglycoside antibiotics: 100% resistance to tobramycin, yet complete susceptibility to gentamicin and amikacin, with no detection of known aminoglycoside-modifying enzyme genes. This phenotype-genotype discrepancy suggests the potential presence of non-classical resistance mechanisms or may be associated with strain-specific genetic backgrounds.

*K. pneumoniae* ST1697 was a novel ST type identified in recent years. Liao et al. (2019) first systematically described ST1697 in their study on carbapenem-resistant *Enterobacteriaceae* colonization in migratory birds at Qinghai Lake, revealing a prevalence of up to 86.7% for this clonal lineage among migratory birds. In this study, sequence analysis confirmed that all five isolates belonged to ST1697, with ANI values ranging from 99.97% to 99.99% confirming their clonal homology. This indicates that the ST1697 clone not only exists in animals but can also cause hospital-acquired infection outbreaks, suggesting its potential for broad adaptability and transmission, which holds significant public health implications. While biofilm-mediated *K. pneumoniae* persistence on endoscopes and tolerance to standard disinfection have been documented previously (Brunke et al., 2022; Cholley et al., 2020), our findings specifically highlight the pediatric intensive care units context and the ST1697 clonal lineage, for which bronchoscope-related nosocomial transmission had not been previously described.

*K. pneumoniae* exhibits extreme resistance in biofilm states, making it difficult to eradicate completely and complicating clinical treatment (Devanga Ragupathi et al., 2020). This study found that the strain isolated from bronchoscope demonstrated the strongest biofilm formation capacity. All strains carried the type 1 and type 3 fimbriae, which are crucial for biofilm formation (Schroll et al., 2010). Once biofilms form, routine cleaning and disinfection procedures often fail to completely eliminate *K. pneumoniae* from bronchoscopes. Arvand et al. (2026) demonstrated that *K. pneumoniae* biofilm exhibited significant tolerance to peracetic acid. Under standard disinfection conditions (0.075% peracetic acid, 37 °C, 5 min), none of the biofilm strains reached the 10^5^ inactivation threshold. The bronchoscope used in this study underwent high-level disinfection according to the Chinese national standard WS 507-2016 (National Health and Family Planning Commission of the People's Republic of China, 2017). The procedure included pre-cleaning, leak testing, washing, rinsing, immersion in Wanjin disinfectant (a chlorine-based disinfectant, 2000 mg/L) for 10 minutes, final rinsing with purified water, drying, and storage. Despite this standardized procedure, CRKP was still isolated. This further demonstrates that biofilms can confer resistance to disinfectants.

Furthermore, the strain isolated from bronchoscope demonstrated the strongest biofilm formation capacity in this study, followed by patient isolates. A possible explanation is that the complex internal structure of endoscopes provides ideal physical conditions for biofilm formation. In contrast, infected tissues within the body exhibit low fluid shear stress and a stable nutrient supply, potentially resulting in weaker selective pressure for biofilm formation compared to medical device surfaces (Cholley et al., 2020). Thus, the strongest biofilm-forming ability observed in bronchoscope isolates also explains why this strain persists despite disinfection.

## 5 Limitations

This study also has certain limitations. First, although clonal homology between bronchoscope strain and patient strains was confirmed, the actual contamination level of bronchoscopes could not be assessed, nor could the actual tolerance level of biofilms to disinfectants be verified by simulating clinical disinfection processes. Second, this single-center report reflects a specific outbreak event; therefore, the generalizability of ST1697 CRKP biofilm characteristics to other settings requires further validation. Nevertheless, based on the combined findings of this study and the previous reports, the risk of bronchoscope serving as vectors for CRKP transmission warrants significant attention (Kakoullis et al., 2024).

## 6 Conclusion

In summary, to our knowledge, this study reports the clonal spread of ST1697 CRKP via bronchoscope in a PICU underscoring the urgency and complexity of preventing bronchoscope-associated infections. These results provide crucial evidence for optimizing bronchoscope reprocessing guidelines and preventing CRKP hospital-acquired infection outbreaks.

## Data Availability

The datasets presented in this study can be found in online repositories. The names of the repository/repositories and accession number(s) can be found in the article/**Supplementary Material**.
