# Supplementary material for: Bronchoscope: an important and easily overlooked vector of clinical infection with Klebsiella pneumoniae
Source: Front Cell Infect Microbiol. 2026 Jun 5;16:1850121. doi: 10.3389/fcimb.2026.1850121 (PMC13279411; doi:10.3389/fcimb.2026.1850121)
Supplement: Supplementary file 1 [file Table1.doc]

Table S1. BioSample accession numbers of the 5 CRKP isolates.

| **Strain ID** | **BioSample Accession** | **Organism** |
| --- | --- | --- |
| KP-2110299021 | SAMN56934591 | *Klebsiella pneumoniae* |
| KP-2111019046 | SAMN56934592 | *Klebsiella pneumoniae* |
| KP-2111039028 | SAMN56934593 | *Klebsiella pneumoniae* |
| KP-2111039005 | SAMN56934594 | *Klebsiella pneumoniae* |
| KP-21800014C | SAMN56934595 | *Klebsiella pneumoniae* |
